# Methods and results from the genome-wide association group at GAW20

**DOI:** 10.1186/s12863-018-0649-0

**Published:** 2018-09-17

**Authors:** Xuexia Wang, Felix Boekstegers, Regina Brinster

**Affiliations:** 1 University of North Texas, GAB 459, 1155 Union Circle #311430, Denton, TX 76203 USA; 2 Institute of Medical Biometry and Informatics, University of Heidelberg, Im Neuenheimer Feld 130.3, 69120 Heidelberg, Germany

**Keywords:** Genome-wide association studies, Phenotype imputation, Empirical kinship matrix

## Abstract

**Background:**

This paper summarizes the contributions from the Genome-wide Association Study group (GWAS group) of the GAW20. The GWAS group contributions focused on topics such as association tests, phenotype imputation, and application of empirical kinships. The goals of the GWAS group contributions were varied. A real or a simulated data set based on the Genetics of Lipid Lowering Drugs and Diet Network (GOLDN) study was employed by different methods. Different outcomes and covariates were considered, and quality control procedures varied throughout the contributions.

**Results:**

The consideration of heritability and family structure played a major role in some contributions. The inclusion of family information and adaptive weights based on data were found to improve power in genome-wide association studies. It was proven that gene-level approaches are more powerful than single-marker analysis. Other contributions focused on the comparison between pedigree-based kinship and empirical kinship matrices, and investigated similar results in heritability estimation, association mapping, and genomic prediction. A new approach for linkage mapping of triglyceride levels was able to identify a novel linkage signal.

**Conclusions:**

This summary paper reports on promising statistical approaches and findings of the members of the GWAS group applied on real and simulated data which encompass the current topics of epigenetic and pharmacogenomics.

## Background

Over the last decade, genome-wide association studies (GWAS) have proven a useful systematic method to investigate the genetic complexities of hundreds of disease phenotypes and their associations with common genomic variations. To date, more than 1000 GWAS have identified more than 4000 significant loci as associated with 500 human diseases and traits [1]. Although GWAS for common variants have thus far achieved substantial success, their findings generally only explain a modest fraction of disease heritability [2, 3]. Potential reasons of missing heritability could be the limited power of GWAS [3] or the contribution of genetic variation such as rare variants [4]. As a consequence of the statistical burden of multiple comparisons in GWAS, reaching the threshold of statistical significance by GWAS can be a challenge. To be considered “‘GWAS significant,” only those associations with a *p* < 5 × 10^− 8^ are considered statistically significant with single-marker analysis [3, 5].

To increase power and interpretability of GWAS, researchers of the Genome-wide Association Study Group of GAW20 have focused on topics such as gene-level association studies for main [6] or gene–environment interaction effects [7]; haplotype-based tests [8]; joint analysis of multiple phenotypes [9]; joint analysis of genetic and epigenetic data [10]; phenotype imputation [11] and employing the empirical kinships in estimating phenotype heritability [12]; genome-wide linkage scan [13]; and genomic prediction of phenotypes [14] (Table 1 and Table 2).

**Table 1:** Contributions toward association tests from the GWAS group

Goal	Reference	Phenotype	Data type	Statistic type
A gene-level association test	Park et al. [6]	Single quantitative trait	Family real GOLDN genetic data	Combined score based test
A haplotype based association test	Zhou et al. [8]	Single binary trait	Case-control and trio GOLDN real genetic data	Bayes factor
A gene-environment interaction test	Gao et al. [7]	Single quantitative or binary trait	GAW20 Case-control simulated genetic data	Score based test
Joint association analysis of single SNPs and DNA methylation markers	Shen et al. [10]	Single quantitative trait	Family real GOLDN genetic and methylation data	Score based test
Jointly analyzing multiple phenotypes	Deng et al. [9]	Multiple quantitative traits	Family real GOLDN genetic data	Pedigree-based USAT (pUSAT)

*GOLDN* Genetics of Lipid Lowering Drugs and Diet Network, *SNP* single-nucleotide polymorphism, *USAT*, unified score-based association test

**Table 2:** Contributions toward phenotype imputation and empirical kinship application from the GWAS group

Contribution	Genotypes	Phenotypes	Evaluation	Quality Control
Chen et al. [11]	Restricted simulated SNP genotypes. 1. Null scenario: 19,763 SNPs on chromosomes 21 and 22 2. Alternative scenario: 5 known causal SNPs (*rs9661059, rs736004, rs1012116, rs10828412* and *rs4399565*)	Simulated TG levels (a) Average difference between pretreatment (visits 1 and 2) and posttreatment (visits 3 and 4) *or* (b) single difference between visits 1 and 3 *of log-transformed TG levels*	1. Type I error rate evaluation in “null scenario” 2. Power evaluation in “alternative scenario”	No quality control (QC) conducted on restricted simulated data
Blackburn et al. [12]	Genome-wide (autosome) SNP data from 822 subjects in 173 pedigrees	TG and HDL-C levels were averaged for pre-treatment (visits 1 and 2) and post-treatment (visits 3 and 4) and regressed on age, sex, their interactions (age × sex, age^2^, age^2^ × sex), study center, smoking, and principal components 1–4; resulting residuals were inverse normalized	Under 3 different kinship models: 1. Heritability analyses 2. Single variant association testing	Exclusion of 6 individuals with unexpected relationships Variants were uplifted to hg19 mapping coordinates, excluding 135 conversion failures
Porto et al. [14]	Genome-wide (autosome) SNP data from 822 subjects in 173 pedigrees	Averaged TG levels of pre- (visits 1 and 2) and post-treatment (visits 3 and 4)	Genomic best linear unbiased prediction (G-BLUP) under 3 different kinship models	Exclusion of 6 individuals with unexpected relationships; variants were uplifted to hg19 mapping coordinates, excluding 135 conversion failures
Peralta et al. [13]	Genome-wide (autosome) SNP data from 822 subjects in 173 pedigrees	Averaged and log-transformed TG levels pre-treatment (visits 1 and 2) and post-treatment and corresponding empirical genetic values (EGVs) from Porto et al. [14] Simulated traits with zero mean, unit variance and a 35% heritability, but not linked to any real loci	Multipoint variance component linkage analyses under the pedigree-based kinship model	Exclusion of 2 individuals with unexpected relationships and 1 monozygotic twin to guard against the artificial inflation of heritability estimates. Variants were uplifted to hg19 mapping coordinates, excluding 135 conversion failures; LD based pruning of r^2^ ≥ 0.9 and exclusion of variants of minor allele count (MAC) > 5 left 375,632 variants for analysis

*HDL-C* high-density lipoprotein cholesterol, *LD* linkage disequilibrium, *SNP* single-nucleotide polymorphism, *TG* triglyceride

Here, we provide a summary of the current literature with respect to methods in GWAS and ways to increase the power of these tests. We then provide contributions from the Genome-wide Association Study Group of GAW20 in the methods and results sections and conclude with recommendations and open problems in the discussion section.

### Current literature on association tests

#### Single-trait association tests

GWAS is considered as the standard approach to detecting common genetic variants associated with complex traits. It is now popular to extend the most popular single-nucleotide polymorphism (SNP)-level analysis to gene-level analysis by aggregating multiple SNPs in a gene, pathway, network, or any region in the genome, such as a haplotype block [15]. SNP-set association tests are believed to be advantageous in several ways [16]. By incorporating linkage disequilibrium and haplotype information among the markers being tested, joint analysis of multiple markers can be more powerful in detecting associated variants with small effects. In addition, the results obtained from SNP-set tests at the gene level can be more readily extended to and integrated with downstream functional and pathogenic investigation because a gene is the basic functional unit of inheritance [17]. As a complement to the standard single SNP-based approach, the gene-level approach can achieve higher reproducibility. Additional benefits of the gene-level approach include a decreased number of hypotheses to be tested, thus a reduced burden of multiple testing.

Several multimarker methods have been proposed based on dimension-reduction techniques, such as principal component analysis [18], partial least-squares regression [19, 20], and methods that are based on combining the *p* values of single-marker tests [21–23]. However, these SNP-set based methods are limited to unrelated samples. Their extensions to incorporate family data may not be feasible. Recently, several methods that are based on a linear mixed model or a generalized linear model have gained increasing popularity [24, 25], such as the kernel machine regression test [26, 27], the sum of squared score (SSU) test [28], the sum of powered score tests [29], variable weight test for testing the effect of an optimally weighted combination of variants (VW-TOW) [30], and haplotype-based logistic Bayesian LASSO (least absolute shrinkage and selection operator) [31]. They provide a flexible and computationally efficient framework for testing the joint effect of SNPs in a SNP set, and have been shown as an attractive alternative to the standard multivariate test under a variety of settings.

#### Multiple traits association tests

Increasing evidence shows that pleiotropy, the effect of 1 variant on multiple traits, is a widespread phenomenon in complex diseases [32]. Furthermore, in genetic association studies of complex diseases, multiple related traits are usually measured. Although most published GWAS analyze each of the related traits separately, joint analysis of multiple traits may increase statistical power to detect genetic variants [33]. Consequently, joint analysis of multiple traits is now popular. Several statistical methods have been developed for joint analysis of multiple traits. These methods can be roughly divided into 3 groups: combining the univariate analysis results [34], regression methods [35–37], and dimension reduction methods [38, 39]. Yang et al. [39] and Ott et al. [38] describe a number of approaches elaborately, including multivariate regression models, variable reduction methods such as principal component analysis, and canonical correlation analysis. However, there is no single approach that is uniformly most powerful across all situations. The sum of squared score (SSU) test does not explicitly incorporate trait correlation, and multivariate analysis of variance (MANOVA) could fail to detect pleiotropy when a strong trait correlation exists and the traits have same direction of association [40]. Considered to be an optimal weighted combination of MANOVA and SSU, the unified score-based association test (USAT) by Ray et al. [40] may provide higher power, especially for detecting pleiotropy.

## Methods

### GAW20 data

The GAW20 data are derived from the Genetics of Lipid Lowering and Diet Network (GOLDN) study, which aims to identify genetic markers of lipid response to fenofibrate treatment. The real data consists of high-density lipid cholesterol (HDL-C) and triglyceride (TG) levels measured before (visits 1 and 2) and after (visits 3 and 4) treatment with fenofibrate in 822 pedigree-based European Americans from 2 different centers in the United States (Minneapolis and Salt Lake City). In GAW20, genome-wide methylation, as well as genome-wide SNP data, from the GOLDN project was made available. Furthermore, simulated post-treatment and pre-treatment TG, methylation levels and SNP genotype data are provided based on the real GOLDN data set. Tables 1 and 2 provide information on the data used in each contribution, whether the data is real or simulated, the phenotype of interest, and the evaluation method used, as well as information on quality control.

The 9 contributions from the GWAS group of GAW20 extend upon the current literature and reflect varied goals, including the creation of new statistic tests, development of phenotype imputation methods, and application of the empirical kinship matrices. Table 1 summarizes contributions that focused on association study and Table 2 summarizes contributions on imputation and empirical kinship estimation.

### New statistics

To perform a gene-level association test to detect genes significantly associated with a single trait using the GAW20 data while effectively controlling for the false-positive rate, Park et al. [6] extended the adaptive sum of powered score (aSPU) test [29], which accounts for unknown and varying association patterns across the genes, thereby maintaining higher power than other nonadaptive gene-level tests. The aSPU test is based on generalized linear models (GLMs). It is computationally feasible as it is not necessary to fit separate models for each SNP or gene, and it is shown to satisfactorily control false-positive rates. To account for individual relatedness and population structures in pedigree data such as GAW20, Park et al. [6] proposed a gene-level aSPU test based on the framework of linear mixed models (LMMs). It is a data-adaptive method that combines the results across a class of score-based tests and only requires fitting a model under the null hypothesis for the whole genome, which makes it computationally efficient.

Zhou et al. [8] proposed an extension of the logistic Bayesian LASSO methodology so that both case-control and trio data can be analyzed jointly in the hope of obtaining an increased statistical power, especially for detecting association between rare haplotypes and complex diseases. The methodology is further extended to account for familial correlation among the case-control subjects and the trios. The authors described the composite likelihood of the whole data as a multiplication between the haplotype-based likelihood for the case-control data and the haplotype-based likelihood for the case-parent trios. However, as a consequence of the complex relationships among the extracted cases, controls, and trios, it is difficult to formulate the correct likelihood. Fortunately, it is possible to obtain correct inferences based on the misspecified composite likelihood through appropriate adjustment. Based on a Bayesian framework, they used the adjusted likelihood for correct inference. The posterior odds over the prior odds, namely the Bayes factor, is used to assess the significance of the coefficients of the genetic terms in the logistic regression model.

Existing methods to detect the main effect of rare variants cannot be readily applied for testing the gene environment interaction effect of rare variants, as those methods either have unstable results or inflated Type I error rates when the main effect exists. To overcome these difficulties, Gao et al. [7] developed a novel score-based test for testing of optimally weighted combinations of SNP environment interaction (TOW-SE) of rare variants. The authors employed a GLM to model the relationship between the trait and gene–environment interactions. They first obtained the residuals of the trait and gene–environment interaction, respectively, by adjusting for covariates. They used the residuals to build a new GLM. They analytically derived a score test with optimal weight for gene–environment interactions to test the TOW-SE. Based on TOW-SE, they proposed a variable weight TOW-SE (VW-TOW-SE) to test gene–environment interaction effects for both common and rare variants.

Advances in high-throughput technologies provide comprehensive assessment of biomarkers, which enable us to systematically study the role of different types of omic data (eg, DNA, DNA methylation) in human diseases. The collection of multilevel omic data from these studies provides us a great opportunity to integrate information from different levels of omic data into association analysis. Although omic-based association analysis holds great promise for discovering novel disease-associated biomarkers, there is a lack of appropriate statistical tools to analyze multilevel omic data [41, 42]. The development of advanced methods to address analytical challenges faced by ongoing omic data analysis can enhance our ability to identify new disease-associated biomarkers. Shen et al. [10] proposed a joint conditional autoregressive model to model the joint effect of genetic markers and DNA methylation on the phenotype of interest. A linear score test is used for hypothesis testing and the corresponding *p* value can be obtained using the Davies method [43].

The true genetic sizes and the direction of associations are usually unknown (a priori) and therefore one would not know which approach is the best for the study. Ray et al. [40] proposed an approach called the USAT to combine MANOVA and SSU. USAT takes the advantages of MANOVA and SSU but does not require the prior knowledge of true effect sizes or correlations among traits. The method was originally designed for independent samples. To account for individual relatedness and population structures in pedigree data, Deng et al. [9] expanded USAT to related samples as a pedigree-based USAT (pUSAT).

### Phenotype imputation

The aim of GWAS is the identification of particular SNPs associated with an outcome of interest, such as the TG or HDL levels in the GAW20 data set. To identify associated SNPs with small and large effect sizes, the power (probability of rejecting a false null hypothesis correctly) of GWAS should be sufficiently high. Missing phenotype data, owing to cost, loss of follow-up or inaccessibility of data, might lead to a decrease of statistical power and consequently to the loss of true SNP associations. A well-known approach to deal with missing data in GWAS is imputation based on available phenotypic data, for instance with methods such as *PhenIMP* [44]. Chen et al. [11] modified this imputation method by including information on family structure, which might lead to higher statistical power in GWAS compared to the consideration of phenotypic data for imputation alone. The information on family structure is derived from the kinship matrix, which might be obtained using the pedigree structure in families or empirical estimations with genotypes. Chen et al. [11] derived a multivariate normal distribution for missing phenotypes with the information on the estimated family structure and additional correlated phenotypes. The expected missing phenotypes were then estimated using the maximum likelihood estimator (MLE) in the SOLAR (Sequential Oligogenic Linkage Analysis Routines) software [45].

### Empirical kinship matrix application

“Kinship” typically refers to the degree of genetic relatedness or coefficient of relationship between individual members of a pedigree. There are at least 2 ways to model kinship of a pedigree: (a) pedigree-based kinship uses specified pedigree relationships, and (b) empirical kinship estimates familial relationships using genomic data. Pedigree-based kinship estimation may be inaccurate or incomplete. Compared to the pedigree-based kinship estimation, the uncertainty surrounding pedigree relationships is reduced with empirical kinship estimates [46] The potential beneficial effect of empirical kinship is investigated by 3 of the GAW20 contributions (eg, Blackburn et al. [12]; Peralta et al. [13]; and Porto et al. [14]) on heritability estimates, genomic predictions, and association and linkage mapping. The software PREST-plus was used to confirm recorded pedigree relationships and examine unexpected relatedness between individuals within and across pedigrees. Inconsistencies were removed for all analyses of the empirical kinship contributions. The remaining pedigree records served to compute the pedigree based kinship estimates with the software SOLAR [46]. Two established methods, LDAK [47] and IBDLD [48], were used to calculate the empirical kinship estimates based on the GAW20 SNP array genotype data.

Phenotypes are influenced by environmental and genetic factors. When it is predicted with genome-wide markers alone, this is called genomic prediction. The resulting empirical genetic value is interpreted as the individual’s phenotype with environmental effects removed. One promising approach for genomic prediction is the genomic–best linear unbiased prediction method (G-BLUP), which uses kinship estimates. With the pedigree and empirical kinships as respective input to G-BLUP, Porto et al. [14] applied the G-BLUP method to the empirical genetic value for each TG phenotype and each individual.

## Results

Park et al. [6] applied the proposed gene-level aSPU approach to test for association with the high-density lipoprotein (HDL) ratio of post-treatment and pre-treatment in GAW20 data. Using the LMM similar to that used by Aslibekyan et al. [49], the proposed method identified 2 nearly significant genes (*APOA5* and *ZNF259*) near rs964184, while none of the other gene-level tests nor the standard test on each individual SNP detected any significant associations in a genome-wide scan.

Zhou et al. [8] used a 2-step strategy to analyze the GAW20 real data. In the first step, they used the Monte Carlo pedigree disequilibrium test to scan the whole genome and determine interesting regions for the adenosine triphosphate binary trait. In the second step, they formed haplotype blocks around the SNPs selected from the first step. They then applied an extension of the logistic Bayesian LASSO to identify haplotypes within each block that have a significant influence on the adenosine triphosphate binary trait. Decision on the significance of a haplotype is based on both Bayes factor (> 2) and confidence interval. Six significantly associated haplotypes were identified (the Bayes factor of the most significant haplotype is 20.7); most are in blocks contained in protein-coding genes that appear to be relevant for metabolic syndrome.

Simulation studies of Gao et al. [7] showed that the 95% confidence interval of the estimated Type I error rates covered the true Type I error. Comparing the 2 methods with the existing interaction sequence kernel association test [50], the VW-TOW-SE was the most powerful test; TOW-SE was the second most powerful test when gene–environment interaction effect exists for both rare and common variants. The proposed tests are applied to the GAW20 simulated data, among the 5 regions, including causal SNPs rs736004, rs1012116, rs4399565, rs9551059, and rs10828412, in which the main effect of common SNPs was included and the gene–age interaction effect was not included. As expected, none of the tests indicate positive results.

The joint conditional autoregressive model of Shen et al. [10] was applied to the GAW20 data from the GOLDN project. In this application, the authors consider a baseline model and a full model. In the baseline model, they considered 3 different scenarios: a model with only genetic information, a model with only DNA methylation information at visit 2, and a model using both genetic and DNA methylation information at visit 2. For the full model, they considered both genetic and DNA methylation information at visit 2 and visit 4. The top 10 significant genes were reported for each model. Based on the results, they found that the gene *MYO3B* was significant when the methylation information was considered in the analysis (*p* value = 0.000759).

Deng et al. [9] applied different approaches to analyze multiple traits (eg, TG and HDL) in the GAW20 real samples and compared the results. Through simulation studies, they confirmed that the Type I error rate of the pUSAT is appropriately controlled. In marginal analysis of TG levels, they found 1 subgenome-wide significant variant on chromosome 6. Joint analyses identified several suggestive genome-wide significant signals on chromosomes 4, 6, and 12 associated with TG and HDL. The pUSAT yielding the greatest number of significant results.

Chen et al. [11] evaluated the proposed approach on complete and incomplete data sets. The incomplete data set corresponded to the simulated data set where samples with at least 1 missing TG value were removed, leading to an incomplete data set of 563 individuals. In contrast, the complete data set contained available TG values and imputed values of missing TG values for a total of 680 individuals. The Type I error rates were evaluated based on the null scenario of 19,763 simulated noncausal SNPs on chromosomes 21 and 22. Statistical power in association analyses were evaluated based on 5 causal SNPs, described in the GAW20 Simulation Solutions. The distribution of missing values is derived using information contained in the missing sample’s relatives and additional correlated phenotypes. They showed that this imputation method can improve power in the association analysis compared with excluding observations with missing data, while achieving the correct Type I error rate.

Blackburn et al. [12] estimated heritability and conducted single-variant association testing using estimates of the pedigree-based kinship and empirical kinship matrices, respectively. The phenotypes under consideration were inverse-normalized residuals of regressed and averaged pre-treatment (visits 1 and 2) and post-treatment (visits 3 and 4) TG and HDL-C levels. Using SOLAR, pedigree-based kinships and empirically calculated kinships (from IBDLD and LDAK) are used to calculate phenotype heritability. In addition, a genome-wide association study was conducted using each kinship model for each phenotype to identify genetic variants significantly associated with phenotypic variation. The variant rs247617 is significantly associated with HDL-C levels both pre-treatment and post-treatment with fenofibrate. Overall, the phenotype heritabilities calculated using pedigree-based kinships or either of the empirical kinships generated using IBDLD or LDAK were comparable. Phenotype heritabilities estimated from empirical kinships generated using IBDLD were closest to the pedigree-based estimations.

Porto et al. [14] studied 2 different factors that influence the prediction of accuracy of G-BLUP for the analysis of human data: (a) the choice of kinship matrix, and (b) the overall level of relatedness. The resulting genetic values represent the total genetic component for the phenotype of interest and can be used, therefore, to represent a trait without its environmental component. Finally, they demonstrated using empirical data how this method can then be used to increase the power of genetic mapping studies.

Peralta et al. [13] (collaborators of Porto et al. [14]) chose the multipoint variance component approach for linkage mapping. Averaged log-normalized TG levels pre-treatment and post-treatment and the corresponding empirical genetic value derived from Porto et al. [14] were considered in the analyses. It was expected that traits, fully explained by available genome-wide markers (ie, with a 100% heritability), will increase the genetic signal in linkage studies. They conducted a genome-wide linkage scan to detect loci that influence the levels of fasting TGs in plasma. Multipoint identity by descent matrices are derived from genotypes using IBDLD. Variance-component linkage analyses were then conducted using SOLAR. They found evidence of linkage (LOD [logarithm of odds] ≥3) at 5 chromosomal regions with TG levels in plasma. Their results suggest that a chromosome 10 locus at 37 cM (LODpre = 3.01, LODpost = 3.72) influences fasting TG levels in plasma regardless of the fenofibrate intervention, and that loci in chromosomes 1 at 170 cM and 4 at 24 cM cease to affect the TG levels when fenofibrate is present, whereas the regions in chromosomes 6 at 136 cM to 162 cM and 11 at 39 cM to 40 cM appeared to influence TG levels in response to fenofibrate.

## Discussion

A central goal of human genetics is to identify genetic risk factors for common, complex diseases such as schizophrenia and Type II diabetes. GWAS that measures and analyzes DNA sequence variations from across the human genome is a valuable effort to identify genetic risk factors for diseases that are common in the population. The ultimate goal of GWAS is to use genetic risk factors to make predictions about who is at risk and to identify the biological underpinnings of disease susceptibility for developing new prevention and treatment strategies.

**Fig. 1:**
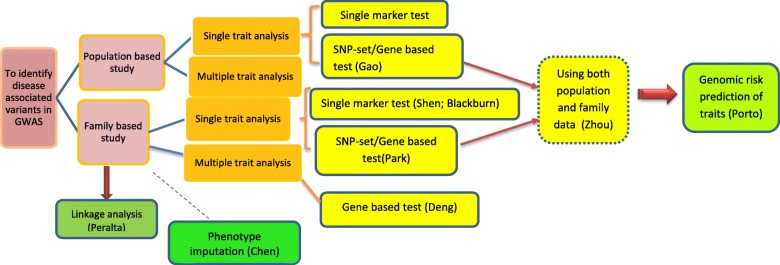
Summary of GAW20 GWAS group contributions

Contributions from the GWAS group of GAW20 provided various statistical approaches which are beneficial in GWAS. Figure 1 summarizes all the contributions of the GWAS group. Population-based study and family-based study are 2 broadly defined study designs employed in GWAS. Gao et al. [7] developed novel tests to detect gene–environment interaction effects using a population-based study design. Shen et al. [10], Blackburn et al. [12], Park et al. [6], Deng et al. [9], Peralta et al. [13], and Chen et al. [11] developed novel methods to detect marginal genetic effect based on family-based design. Zhou et al. [8] developed a novel approach to test for marginal genetic effects by using both population-based case-control data and family-based trio data. For a fixed genotyping budget, population-based design is often the most powerful study design [51]. It is generally believed that family-based design is robust against spurious association because of population stratification or admixture [52].

In genetic association studies of complex diseases, multiple related traits are usually measured. For example, correlated TG and HDL are provided in the GAW20 data, hypertension is evaluated using systolic and diastolic blood pressures, metabolic syndrome is based on observing 3 of 5 criteria [53], and there are highly correlated lipids traits TG and HDL. Although most published GWAS analyze each of the related traits separately, the joint analysis of multiple traits can not only increase statistical power to detect genetic variants [37, 39], but can also be crucial for understanding the genetic architecture of the disease of interest [54]. Consequently, the joint analysis of multiple traits has become popular. Deng et al. [9] extend the USAT to related samples as a pUSAT by incorporating family structure. pUSAT provided comparable results with slightly smaller *p* values than the existing methods when applied to the GAW20 data. Therefore, when there are multiple traits available, we suggest jointly analyzing multiple traits, which can increase both the power and the interpretability of the findings.

The genetic variants discovered by GWAS account for only a small portion of the heritability of complex traits [55, 56]. One possible explanation for the missing heritability is that the analysis strategy commonly used in GWAS, testing for association of the phenotype with each SNP individually, is not well suited for detecting multiple variants with small effects [57]. Proposed research strategies to uncover this missing heritability include studying rare variants such as the TOW-SE proposed by Gao et al. [7], or epigenetic effects such as with the score test developed by Shen et al. [10]. Advanced analyses of GWAS data using novel statistical methods such as gene-set (SNP-set or network-assisted) analysis also have been proposed as a way to extract additional information from genome-wide SNP data [58]. Gene-set analysis aims to assess the overall evidence of association of variation in an entire set of SNPs or genes with a phenotype. The gene set can be defined using canonical pathways [59] gene ontology categories [60], or subnetworks. Pathway-based analysis is 1 type of gene-set analysis that uses canonical pathways, gene ontology biological process categories, or other pathway annotations as its gene-set unit. Gene set has the potential to detect subtle effects of multiple SNPs in the same gene set that might be missed when assessed individually [61]. Because numerous genes can be combined into a limited number of gene sets for analysis, the multiple testing burden may be greatly reduced by gene-set analysis. Moreover, the incorporation of biological knowledge in the statistical analysis may aid researchers in the interpretation of results [62].

To increase the power of a gene-set–based test, many weighting strategies have been proposed [63]. Gao et al. [7] analytically derived optimal weights for TOW-SE to detect gene–environment interaction for rare variants. The assumption of TOW-SE is the independence between variants, which usually holds for rare variants, it needs to explore a more flexible form of the statistic when it is used to common variants. Park et al. [6] proposed a gene-level association test that accounts for individual relatedness and population structures in pedigree data in the framework of LMMs. This method is based on a class of the sum of powered score tests indexed by a positive integer ɣ. Park et al. [6] suggested to treat ɣ as a factor that decides the weight on each score element. If the test statistic could be treated as a function of ɣ, a further work might be done to find the optimal ɣ where the test statistic reaches its maximum. Zhou et al. [8] proposed an extension of the logistic Bayesian LASSO methodology to jointly analyze both case-control and trio data. This is a haplotype-based approach that needs phased haplotypes. Therefore, to ease computational burden, this method should be used on specific genetic regions rather than the whole genome.

GWAS have discovered hundreds of common genetic variants associated with multifactorial diseases. These variants can be added to classical clinical and environmental risk factors for the improvement of risk-prediction assessment. However, for most common diseases, the addition of genetic variants to traditional risk factors has produced only modest improvements [64, 65]. The subsequent genetic risk profiles generated are still unlikely to provide sufficient discrimination to warrant individualized prevention. Porto et al. [14] show that the G-BLUP methods borrowed from animal breeding can be employed to increase the accuracy of genomic prediction of complex phenotypes and the power of genetic mapping studies.

## Conclusions

In summary, the contributions from the GWAS group of the GAW20 provide useful tools for genetic association studies regarding to single variant single-trait analysis [12], gene-based single-trait analysis [6, 8], gene-based joint analysis of multiple traits [9], gene-based gene–environment interaction analysis [7], and joint analysis of genetic and epigenetic effect [10]. Moreover, phenotype imputation technology developed by Chen et al. [11] could be a useful tool to increase sample size and eventually increase power of a test. All the significant genetic variants identified with the aforementioned methods could be used in building risk-prediction models [14] to predict the disease risk of an individual in the general population for a given disease. A well-established prediction model would greatly benefit patients, clinicians, and researchers because it would allow individuals at high risk to be identified at the earliest stage. Early stage detection would be very helpful in reducing disease related morbidity and mortality because treatment might be most effective at the earliest stages of most of the diseases.

## Abbreviations

ASPU test: Adaptive Sum of Powered Score test
G-BLUP: Genomic-best Linear Unbiased Prediction Method
GLMs: Generalized Linear Models
GOLDN: Genetics of Lipid Lowing Drugs and Diet Network
GWAS: Genome-wide Association Study
HDL: High-density Lipoprotein
HDL-C: High Density Lipid Cholesterol
LASSO: Least Absolute Shrinkage and Selection Operator
LMMs: Linear Mixed Models
MANOVA: Multivariate Analysis of Variance
MLE: Maximum Likelihood Estimator
pUSAT: Pedigree-based Unified Score-bases Association Test
SNP: Single Nucleotide Polymorphism
SOLAR: Sequential Oligogenic Linkage Analysis Routines
SSU: Sum of Squared Score test
TG: Triglyceride
TOW-SE: Testing of Optimally Weighted combinations of SNP Environment interaction
USAT: Unified Score-based Association Test
VW-TOW: Variable Weight test for Testing the effect of an Optimally Weighted combination of variants
